# Advances in Point-of-Care Infectious Disease Diagnostics: Integration of Technologies, Validation, Artificial Intelligence, and Regulatory Oversight

**DOI:** 10.3390/diagnostics15222845

**Published:** 2025-11-10

**Authors:** Moustafa Kardjadj

**Affiliations:** CRO Division, Dicentra, Toronto, ON M4W 3E2, Canada; moustafa@dicentra.com or drkardjadj@live.fr

**Keywords:** point-of-care diagnostics, infectious diseases, molecular diagnostics, immunoassays, biosensors, microfluidics, CRISPR-based diagnostics, artificial intelligence, machine learning, regulatory frameworks, CLIA waiver, analytical and clinical validation, post-market surveillance, public health impact, antimicrobial stewardship, global health, digital surveillance

## Abstract

Point-of-care (POC) infectious disease diagnostics are reshaping global health by delivering rapid, decentralized, and clinically actionable results that link bedside testing to population-level surveillance. Valued at approximately USD 53 billion in 2024 and projected to nearly double by 2033, the global POC diagnostics market is driven by infectious disease assays and accelerated by innovations in molecular amplification, biosensors, microfluidics, and artificial intelligence (AI). This review integrates current evidence across technological, clinical, regulatory, and public health domains. Immunoassays remain the backbone of volume deployment, while molecular nucleic acid amplification tests (NAATs) and emerging CRISPR-based platforms achieve laboratory-grade sensitivity at the point of care. AI has transitioned from an experimental tool to an embedded analytical layer that enhances image interpretation, multiplex signal deconvolution, and automated quality control. Rigorous validation, including analytical accuracy, clinical performance in intended-use settings, and usability testing under CLIA guidance, remains central to ensuring reliability in decentralized environments. Regulatory frameworks are adapting in parallel: FDA’s lifecycle oversight of AI-enabled devices, the European IVDR’s expanded evidence requirements, and the WHO Prequalification all emphasize continuous post-market surveillance. From a public health perspective, POC diagnostics have improved early case detection, treatment initiation, and outbreak containment for HIV, tuberculosis, malaria, influenza, RSV, and COVID-19. Yet persistent challenges (including limited harmonization of standards, uneven reimbursement, and scarce real-world data from low- and middle-income countries) continue to constrain equitable adoption. POC infectious disease diagnostics are thus entering a pivotal phase of digitization and regulatory maturity. Addressing remaining gaps in validation, lifecycle monitoring, and implementation equity will determine whether these technologies achieve their full promise as clinical accelerators and as cornerstones of global infectious disease preparedness.

## 1. Introduction

Infectious diseases remain among the leading global causes of morbidity and mortality, accounting for millions of deaths annually despite advances in therapeutics and preventive interventions [1]. Rapid and accurate diagnosis is essential for effective patient management, antimicrobial stewardship, and outbreak control. However, traditional laboratory-based diagnostics are often centralized, infrastructure-dependent, and time-consuming, leading to diagnostic delays that undermine patient care and public health responses [2].

Point-of-care (POC) diagnostics, defined as diagnostic tests performed near the site of patient care with rapid turnaround times, have emerged as transformative tools in this landscape. Their ability to deliver actionable results within minutes to hours enables immediate treatment decisions, reduces loss-to-follow-up, and facilitates decentralized testing in both high- and low-resource settings [3,4]. Early adoption of lateral flow assays (LFAs) for malaria and HIV demonstrated their value in global health programs, while the COVID-19 pandemic accelerated the widespread use of antigen and molecular POC platforms at home, in clinics, and in community settings [5,6].

Recent technological advances (including microfluidics, biosensors, isothermal amplification, CRISPR-based assays, and artificial intelligence (AI)-enhanced platforms) are further expanding the capabilities of POC diagnostics [7,8]. These innovations are coupled with digital connectivity and cloud-based reporting, enabling integration with electronic health systems and near real-time surveillance that can inform outbreak response and resource allocation [9,10]. At the same time, regulatory frameworks such as the U.S. Food and Drug Administration (FDA) 510(k) and CLIA-waiver pathways, Health Canada Medical Device Licensing (MDL), the European Union’s In Vitro Diagnostic Regulation (IVDR), and the WHO Prequalification program are evolving to accommodate the unique features of decentralized testing and AI/ML components [11,12,13].

Despite clear benefits, POC platforms face persistent trade-offs and deployment barriers. Many rapid formats (notably some lateral-flow antigen tests) have lower analytical sensitivity than centralized laboratory NAATs, creating a tension between speed and analytical performance that must be managed through appropriate use-case selection, reflex testing pathways, and quality-assurance programs [6,14]. Device simplicity and automation reduce operator dependence, but robustness in uncontrolled settings requires rigorous human-factors validation and real-world performance data, elements that are increasingly required in regulatory submissions for CLIA waiver and home-use claims [15,16,17].

Beyond infectious disease targets, transferring POC approaches to non-communicable disease biomarkers (e.g., cancer protein panels or complex metabolomic signatures) presents further technical hurdles: these analytes often demand higher analytical precision, multi-analyte standardization, and complex pre-analytic handling that currently favor centralized laboratory platforms [18]. Accordingly, the bulk of POC innovation to date has focused on infectious disease targets where actionable binary or semiquantitative outputs (positive/negative, antigen level above threshold) directly inform immediate clinical decisions.

Finally, market and policy conditions shape the real-world impact of POC diagnostics. Rapid tests only translate into improved health outcomes when linked to clinical guidelines, reimbursement pathways, and data systems that allow case reporting and surveillance [19,20]. For POC devices to fulfill their public health promise (from antimicrobial stewardship to outbreak control), developers must align engineering, clinical validation, regulatory strategy, and implementation science from early development onward. This review synthesizes available evidence on market trends, technology platforms, validation and regulatory pathways, and public health implications, with special attention to AI integration. We conclude by identifying gaps and actionable recommendations to accelerate safe, equitable, and effective POC deployment worldwide.

## 2. Market Analysis

The global point-of-care (POC) diagnostics market was valued at approximately USD 53.1 billion in 2024, with commercial forecasts projecting growth to roughly USD 89–102 billion by the early 2030s [21,22,23,24]. North America accounts for a substantial share of this market (driven by strong R&D investment, widespread adoption of rapid tests across clinical settings, and growth in at-home testing models), while the U.S. alone represented roughly USD 13.1 billion in 2024 (≈24–25% of the global market) [21]. The COVID-19 pandemic accelerated acceptance and scale-up of decentralized testing, demonstrating both the feasibility and the system-level value of massively deployed antigen and molecular POC tests [23,25].

Within the POC diagnostics sector, infectious disease testing forms a distinct and sizable submarket; several market reports place global infectious-POC revenue in the order of USD 12–15 billion for 2024, with multi-year growth in the high single digits under typical scenarios [21,24]. However, near-term realization of projected growth will depend strongly on reimbursement rules, procurement mechanisms, and operational readiness at the point of care; without aligned payment models and supply-chain stabilization, adoption of higher-cost molecular and cartridge-based systems will remain constrained.

### 2.1. Segmentation by Disease

**Figure 1:**
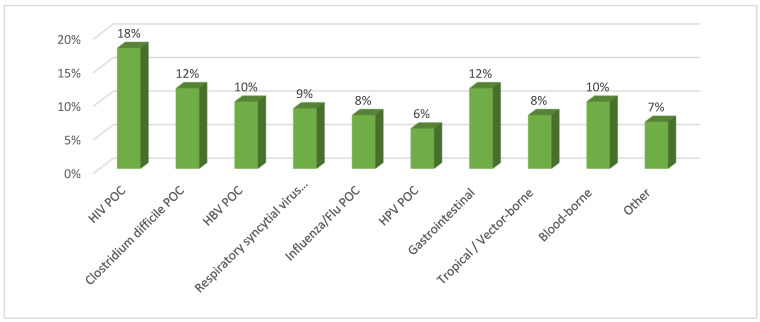
**Global infectious disease POC diagnostics market by disease type (2024 estimates).** HIV (18%), Clostridium difficile (12%), Hepatitis B (10%), RSV (9%), Influenza (8%), HPV (6%), Gastrointestinal pathogens (12%), Tropical/vector-borne diseases (8%), Blood-borne infections (10%), and Other (7%). Percentages derived from published market analyses [19,20,21,22].

Infectious disease POC diagnostics form a sizable and distinct submarket. Reports estimate this segment at USD ~12–15 billion in 2024, with a compound annual growth rate (CAGR) of ∼8–9% through the 2020s [21,24]. Within infectious POC diagnostics, HIV testing leads (18% market share), followed by Clostridium difficile (12%), hepatitis B (10%), respiratory syncytial virus (RSV, 9%), influenza (8%), human papillomavirus (HPV, 6%), gastrointestinal pathogens (12%), tropical/vector-borne diseases (8%), blood-borne infections (10%), and others (7%) [19,20,21,22]. (See Figure 1.) Programmatic screening initiatives and donor-funded programs continue to support scale in HIV and malaria diagnostics and in many antenatal screening use cases; at the same time, tropical and vector-borne disease diagnostics and multiplex gastrointestinal testing are expanding interest in LMIC settings as surveillance and procurement priorities evolve [1,3,9].

### 2.2. Segmentation by Technology

**Figure 2:**
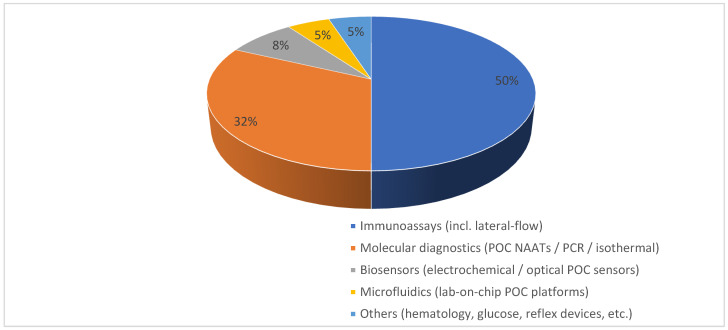
**Global infectious disease POC diagnostics market by technology segmentation (2024 estimates).** Immunoassays (50%), Molecular diagnostics (32%), Biosensors (8%), Microfluidics (5%), and Others (5%). Data synthesized from commercial market reports and peer-reviewed reviews [19,20,21,22].

The infectious disease POC diagnostics market can also be segmented by technology platform, as shown in Figure 2. Immunoassays (including lateral flow and reader-assisted FIA) ≈ 50%, Molecular diagnostics (POC NAAT/PCR/isothermal) ≈ 32%, Biosensors ≈ 8%, Microfluidics ≈ 5%, and Others (hybrid CRISPR, connected readers, niche analyzers) ≈ 5% [19,20,21,22]. Immunoassays maintain a dominant position because of low unit cost, simple user workflows, and regulatory familiarity (many CLIA-waived and OTC options), which make them the first line for large-volume screening and low-acuity testing. Molecular platforms are the fastest-growing technology group because they deliver higher analytical sensitivity and multiplexing capacity; however, uptake into high-volume screening remains tempered by per-test consumable costs, reimbursement barriers, supply-chain considerations, and the need for additional human-factors evidence for broad CLIA waiver or home-use claims [11,12,13].

Biosensors and microfluidic lab-on-chip platforms represent smaller but strategically important growth areas. Biosensor systems can provide quantitative outputs and are particularly well suited to smartphone integration and AI-based signal processing; successful commercialization will require robust calibration, reproducible manufacturing, and validated software pipelines to ensure clinical reliability and regulatory compliance. Microfluidic devices offer multiplexed, sample-to-answer automation, but broad deployment depends on scalable production methods (e.g., transition from prototyping to injection molding or roll-to-roll fabrication), standardized quality control, and reliable handling of complex sample matrices. If manufacturing and standardization challenges are addressed, these platforms could materially increase their market share by delivering low-touch, multiplexed testing in decentralized settings [26,27,28,29].

In sum, the market outlook is mixed but positive: immunoassays will continue to serve as a high-volume backbone for inexpensive screening, while molecular, biosensor, and microfluidic technologies expand in clinical niches where higher sensitivity, multiplexing, or connectivity yield measurable clinical or operational value. The relative pace of change will be determined principally by per-test economics, reimbursement policy, supply-chain maturity, and regulatory clarity for connected and AI-enabled devices [21,22,23,24,25,26,27,28,29]. Policymakers and developers who align price, clinical-utility evidence, and implementation planning will be best positioned to translate technological advantages into sustained market share.

## 3. AI Integration in POC Infectious Disease Diagnostics

Artificial intelligence (AI) is emerging as a transformative overlay across point-of-care (POC) diagnostic technologies, rather than as a distinct technology category of its own. Recent systematic reviews indicate that AI is increasingly being integrated into POC devices for applications such as signal or image interpretation, automated quality control (QC), and point-of-care decision support [30]. Evidence from diagnostic imaging and pathology shows the potential of AI to improve accuracy and reduce inter-operator variability, and analogous benefits are being realized in POC contexts as edge and phone-based models mature [31].

POC-specific AI solutions are gaining momentum. Edge- and smartphone-based AI applications are being developed for low-resource environments, enabling near real-time interpretation of lateral-flow assays and biosensor signals without dependence on central laboratory infrastructure [32]. Pilot deployments in infectious disease screening and decentralized monitoring suggest improved result consistency and shortened time-to-action when AI-assisted readers or apps are used. Importantly, validation of AI models in POC settings requires datasets representative of intended use conditions (diverse populations, variable lighting/temperature, and operator skill levels) to avoid algorithmic bias and ensure generalizability [30,32].

AI should be conceptualized as an enabling layer that cuts across existing technology stacks (Figure 2). A portion of devices within each category (immunoassays, molecular diagnostics, biosensors, and microfluidics) will adopt AI components either via on-device (edge) algorithms or cloud-assisted analytics. Market syntheses and vendor pipelines suggest modest AI penetration in 2024 but substantial uptake by 2034, especially in data-rich platforms such as molecular, biosensor, and microfluidic systems (Table 1) [33].

For immunoassays, the primary benefit of AI is to convert qualitative or visually interpreted strip results into robust, quantitative or semi-quantitative outputs and to automate QC; this capability hinges on a widespread technological shift from unaided visual readers to standardized digital readers or smartphone-imaging workflows. AI-assisted readers can (a) reduce inter-operator variability, (b) detect weak positive lines missed by the human eye, (c) flag invalid tests, and (d) geo-tag and securely transmit test results for surveillance in near real time [30,32]. The prerequisite technological shift is therefore the routine deployment of reader hardware or validated smartphone imaging protocols that standardize image capture (illumination, distance, white balance) and allow algorithms to operate on consistent inputs.

Molecular diagnostics are already software-enabled, which makes them amenable to earlier and deeper AI integration. AI for molecular POC platforms can accelerate result interpretation (e.g., curve/trace analysis and multiplex deconvolution), perform on-board QC and anomaly detection, and optimize instrument workflows; substantial AI penetration in molecular platforms is therefore expected earlier than in reader-less immunoassays. This advantage derives from (a) structured, high-signal data streams (fluorescence curves, time-series traces), and (b) existing digital interfaces that permit controlled model deployment and updates [30,33].

Projected timelines for meaningful AI uptake are heterogeneous across technology classes. Based on current literature, product pipelines, and market analyses, modest AI-enabled adoption (single-digit percent of installed/active products) is observable in 2024, with acceleration through the late 2020s as validated reader hardware, regulatory clarity, and reimbursement pathways mature. By ~2030–2032, we expect mid-range AI penetration in biosensor and molecular categories, and a larger cumulative penetration by 2034 (see Table 1 projections: immunoassays 3% → 15%; molecular 7% → 35%; biosensors 8% → 40%; microfluidics 10% → 45%). These modeled timelines reflect both technological readiness and the regulatory/market factors that influence adoption velocity [30,33].

Biosensors and microfluidic devices are particularly well aligned with AI because they generate high-dimensional or continuous data streams that benefit from denoising, pattern recognition, and predictive analytics (for example, signal deconvolution, baseline drift correction, or multi-analyte pattern detection). AI can therefore increase the clinical utility of these platforms by extracting diagnostically relevant features from noisy raw signals and by enabling predictive use-cases (e.g., deterioration risk flags) when combined with contextual patient metadata [33].

While AI promises to raise performance standards across POC categories, it also introduces new validation and governance needs. Algorithmic bias, model drift in new populations or changing pathogen landscapes, and cybersecurity & data privacy concerns require lifecycle-oriented regulatory and post-market surveillance approaches. Robust clinical/field validation of AI components, ideally using prospective, representative datasets, and transparent reporting of training/validation cohorts are essential preconditions for safe deployment [23,30].

In summary, AI is an enabling layer that will progressively augment immunoassays, molecular platforms, biosensors, and microfluidics, improving accuracy, standardizing interpretation, and adding connectivity & QC features. The pace and depth of integration will vary by technology class, with molecular, biosensor, and microfluidic platforms expected to see the most substantial uptake by 2034, conditional on regulatory clarity and economic incentives.

## 4. Point-of-Care Infectious Disease Diagnostic Technologies

POC infectious disease diagnostics are built on diverse technological platforms, each with distinct detection principles, workflows, and use cases. Table 2 provides a comparative summary of major technology classes, representative commercial devices, and their respective strengths and limitations. Table 2 includes up-to-date example devices and typical performance characteristics. The following subsections summarize the principal platform types, note current commercial examples and regulatory status where relevant, and highlight recent innovations that are most relevant to infectious disease POC applications [34,35,36,37,38,39,40,41].

### 4.1. Immunoassays

Lateral-flow immunoassays (LFIAs) represent the archetypal format for rapid POC infectious disease testing and remain the most widely deployed POC technology due to their simplicity, low per-test cost, and ease of use [34,35]. LFIAs were first widely commercialized in the 1980s (Group A Streptococcus) and today underpin many antigen and antibody rapid diagnostic tests for HIV, malaria, influenza, and SARS-CoV-2 [4,34,36]. These devices typically run in 10–30 min and can be visually read or interpreted using inexpensive portable readers. Because many LFIAs have simple operation and low user-training requirements, they are well suited to community, pharmacy, and at-home use, and many products benefit from CLIA-waived or OTC authorizations in the U.S. [4,34,36].

Recent innovations: efforts have focused on improving analytical sensitivity, quantitative readout, and multiplexing. Nanoparticle-based signal enhancers (fluorescent nanodots, upconverting phosphors, SERS tags) and reader-assisted chemistries increase signal intensity compared with colloidal gold, enabling lower limits of detection and semi-quantitative outputs [37]. Hybrid multimodal LFIAs and “lab-on-strip” formats combine colorimetric, fluorescent, and magnetic labels to expand dynamic range and multiplex capability. Smartphone imaging and AI-assisted interpretation are emerging as practical enhancements that (a) standardize reads across users and environments, (b) reduce false negatives from faint lines, and (c) enable secure, geotagged reporting for surveillance, conditional on validated image capture protocols and regulatory acceptance [37,38].

Limitations: despite ubiquity, LFIAs generally show lower analytical sensitivity than NAATs at low pathogen loads, and performance can be strongly affected by specimen quality and timing of sampling relative to the onset of infection [4,36]. For high-consequence decisions (e.g., treatment initiation for invasive infections), LFIA negatives may require reflex testing with more sensitive methods.

### 4.2. Molecular Diagnostics

Nucleic acid amplification tests (NAATs) at the POC include rapid PCR and isothermal amplification systems. Several cartridge-based platforms (e.g., Cepheid Xpert, Roche Liat, Abbott ID NOW, Visby) deliver sample-in/answer-out workflows with run times typically between ~13 and 60 min and have been granted regulatory clearance and, in many cases, CLIA waiver for specific indications [39,40]. POC NAATs provide laboratory-comparable sensitivity and specificity for many viral and bacterial pathogens, enabling rapid clinical decision-making in EDs, urgent care, and decentralized clinics [39,40].

Recent innovations: CRISPR-Cas diagnostics (SHERLOCK, DETECTR) and related approaches now demonstrate single-molecule sensitivity in simplified formats and can be adapted to lateral-flow readouts [41]. Microfluidic sample-in/answer-out cartridges with lyophilized reagents reduce cold-chain dependence and simplify field deployment [41,42]. Advances in microheater designs and ultrafast thermal cycling are shortening PCR run times, and portable isothermal platforms extend molecular testing to near-patient and some home contexts [42,43].

Limitations: cartridge costs, single-use consumable economics, supply chain dependencies, and device maintenance requirements limit immediate displacement of low-cost immunoassays in many settings. Consequently, molecular POC adoption is concentrated where clinical value (rapid pathogen ID, antimicrobial guidance, or urgent infection control actions) offsets higher per-test cost [39,40].

### 4.3. Biosensors

Biosensors transduce a specific biorecognition event (antibody, aptamer, nucleic acid probe) into an electrical, optical, or mechanical signal. Electrochemical sensors, optical SPR/SERS chips, and other reader-based platforms can achieve rapid, and in some cases quantitative, detection from small sample volumes (fingerstick blood, saliva) [44,45]. Biosensors are attractive for POC settings because they can deliver high temporal resolution data, are amenable to miniaturization, and readily pair with smartphone or dedicated readers for AI-assisted interpretation and connectivity [44].

Recent innovations: incorporation of advanced nanomaterials (graphene, magnetic nanoparticles, quantum dots) has improved capture efficiency and signal-to-noise ratios [46]. Smartphone-coupled biosensors leverage built-in optics and processing for on-device analysis and secure data transfer [47]. Wearable biosensors and IoMT-linked devices are under development for continuous or near-continuous infection surveillance.

Limitations: fewer biosensor platforms have completed large-scale clinical validation or achieved broad regulatory clearance for infectious disease indications compared with LFIA and NAAT systems; common challenges include calibration drift, batch-to-batch variability, and integration of robust sample preprocessing for complex specimen matrices [44,46].

### 4.4. Microfluidics

Microfluidic technologies miniaturize laboratory workflows on-chip, enabling automated sample metering, reagent handling and multiplex detection within sealed cartridges [48]. Many current cartridge NAAT platforms rely on underlying microfluidic principles to achieve closed workflows (e.g., Xpert, Liat) [39]. Microfluidic lab-on-chip systems promise high degrees of integration (sample-in/answer-out), minimal operator steps, and multiplexing capacity, attributes highly valuable for decentralized infectious disease testing [48].

Recent innovations: paper-based microfluidics (foldable “origami” devices), centrifugal microfluidics (“lab-on-a-disc”), capillary-driven self-powered chips, and 3D-printed cartridges lower cost and complexity for some applications [45,49]. Multiplex microfluidic chips capable of simultaneous detection of respiratory, enteric, or STI panels are advancing toward field trials.

Limitations: scaling manufacturing (transition from soft lithography to injection molding/roll-to-roll production), standardizing materials and QC, and ensuring robust operation across diverse specimen types and environmental conditions remain major translational hurdles, factors that currently constrain widespread market penetration despite technical promise [48,49].

### 4.5. Other Emerging Technologies

Beyond established platforms, several novel POC technologies are being developed. CRISPR-based strip assays combine nucleic-acid specificity with LFIA usability, bridging molecular and immunoassay formats [47]. Portable sequencing devices such as Oxford Nanopore MinION enable on-site genomic surveillance of pathogens, while smartphone-based microscopes and fluorescence readers expand the accessibility of advanced assays [48].

Recent innovations emphasize integration with AI and connectivity. Deep learning is increasingly applied for faint-line interpretation and automated reporting, while blockchain-backed platforms ensure secure transmission of diagnostic data to public health networks [49,50]. Wearable devices and fully integrated digital POC ecosystems, where sampling, analysis, and reporting are managed by smartphone-guided cartridges, represent the next frontier.

## 5. Validation of POC Infectious Disease Diagnostics

Rigorous validation of point-of-care (POC) diagnostics is essential to ensure accuracy, reliability, and clinical usefulness across diverse healthcare environments. Unlike centralized laboratory assays, POC devices are often used by non-specialists (e.g., nurses, community health workers, or patients) in uncontrolled settings. This reality underscores the importance of multi-dimensional validation that encompasses analytical accuracy, clinical performance in intended-use settings, and human-factors/usability testing. Validation must therefore demonstrate not only analytical equivalence to accepted reference methods but also operational fitness for the intended user, setting, and clinical algorithm [51,52,53].

Analytical validation is the foundation of regulatory review and device claims. It includes determination of the limit of detection (LOD), analytical specificity (cross-reactivity), interference testing, dynamic range, precision (repeatability and reproducibility), linearity, and stability under expected storage and use conditions. For semi-quantitative assays, comparison methods such as receiver operating characteristic (ROC) analysis, Bland–Altman plots, Passing–Bablok regression or Deming regression are used to evaluate agreement with a laboratory reference method [54]. Robust analytical validation should explicitly report the specimen types, matrix effects, and any pre-analytic steps (e.g., dilution, extraction) required to achieve the reported performance [52,53,54].

Clinical performance refers to how the test performs on real patient specimens in the intended clinical environment. Clinical performance evaluations typically involve prospective, multicenter studies that compare the POC device to a laboratory “gold standard” (for example, laboratory RT-PCR for viral detection), reporting sensitivity, specificity (or PPA/NPA), and predictive values in the target population [55]. Clinical performance studies must be designed with representative prevalence, specimen collection practices, and operator profiles to ensure generalizability; single-site or convenience sample studies are insufficient to support wide deployment [55].

Observed variability across studies. Although some POC molecular platforms have demonstrated very high performance in rigorous multicenter evaluations (for example, the Cue Health COVID-19 molecular POC study reporting 100% sensitivity and 99.4% specificity versus laboratory RT-PCR in a primarily asymptomatic cohort [56]), it is critical to avoid broad generalization from a single study. Performance of POC platforms may vary by assay version, specimen type, user, viral load distribution and study design; evaluations of other POC isothermal or LAMP assays have, in some cases, reported lower sensitivity relative to laboratory RT-PCR in certain cohorts or specimen types. This heterogeneity underscores the need for multiple, independent clinical evaluations across intended use settings before assuming consistent, PCR-comparable performance for all POC NAATs [53,54,55,56].

Human factors and usability validation are increasingly required in regulatory submissions, particularly for CLIA-waiver or OTC/home-use claims. Human-factors studies (simulated use and formative/summative usability tests) assess whether intended users can perform the test correctly and interpret results consistently without specialized laboratory training. Usability studies should include diverse operator types, common user errors, and environmental extremes (temperature, humidity, vibration) where appropriate, and should quantify user error rates and invalid test frequencies. Many regulators (including the FDA) expect such data for waiver or home-use consideration, and these studies often form a decisive part of the risk/benefit assessment [57].

Post-market and real-world validation. Given the risk of performance drift over time (manufacturing lot variability, reagent stability, pathogen evolution), regulators and purchasers increasingly rely on post-market surveillance, external quality assessment (EQA) programs, and real-world evidence to confirm ongoing device performance. Manufacturers should plan prospective post-market studies, mechanisms for rapid field feedback (e.g., connected device telemetry), and participation in EQA/PT schemes to detect batch or field-use problems early [52,53,54,55].

Table 3 summarizes representative FDA-cleared/CLIA-waived POC infectious disease diagnostics and illustrates the range of technologies and typical performance claims in manufacturer IFUs and published evaluations. When summarizing such data in tables or text, qualitative labels (e.g., “High,” “Moderate”) are intended as concise summaries; investigators and clinicians should consult IFUs and peer-reviewed comparative studies for exact PPA/NPA and LOD values in the relevant specimen types and populations [52,53,54,55,56].

Common validation weaknesses. A persistent shortcoming in POC validation is the reliance on single-site, convenience cohorts, or small sample sizes that do not capture user variability, population diversity, or real-world pre-analytic variability. To support regulatory claims and clinical adoption, developers should prioritize multicenter prospective studies, external validation by independent laboratories, robust human-factors testing, and pre-planned post-market surveillance [52,53,54,55].

## 6. Regulatory and Implementation Considerations

### 6.1. Regulatory Pathways

In the United States, point-of-care (POC) infectious disease diagnostics are regulated as in vitro diagnostics (IVDs) by the FDA and cleared via 510(k) (Class II), De Novo, or PMA (Class III) pathways, depending on risk category and intended use [57,58,59,60]. Importantly, many POC assays also pursue CLIA-waived status, which permits use outside high-complexity laboratories. To obtain a waiver, manufacturers must demonstrate that the device is simple to use and carries an “insignificant risk of erroneous result” even under challenging environmental or operator conditions; this typically requires human-factors studies, robustness testing, and fail-safe mechanisms [61,62]. Tests cleared for home/OTC use are automatically eligible for CLIA waivers, which materially expands access but also places a premium on usability and clear labeling [61,62].

POC tests intended for global markets must navigate other regulatory frameworks. Health Canada regulates IVDs under a risk-based system (Class I–IV) and requires a Medical Device Licence (MDL) for Class II+ devices [63]. The European Union’s IVDR (Regulation 2017/746) places many infectious disease POC tests in Class B or C, requiring conformity assessment by a Notified Body and robust clinical performance evidence [64]. WHO Prequalification of Diagnostics (PQDx) evaluates tests for procurement by UN and donor programs, emphasizing Target Product Profiles (TPPs), ease of use, and field performance for low-resource settings [65]. International harmonization efforts (IMDRF, WHO’s Global Model Regulatory Framework) are active, but important differences remain in classification granularity, clinical-evidence expectations, and post-market obligations; developers should therefore plan region-specific evidence packages early in development [66].

Table 4 (below) provides a side-by-side comparison of major regulatory regimes, highlighting differences in device classification, analytical/clinical evidence requirements, human-factors expectations, post-market surveillance, and typical timelines for POC IVDs [57,58,59,60,61,62,63,64,65,66,67].

### 6.2. AI-Enabled Diagnostics

Regulatory expectations for AI/ML in IVDs are evolving rapidly, and sponsors of AI-enabled POC devices must plan for a lifecycle approach to validation and oversight. The FDA and other authorities expect evidence of algorithm training, performance across diverse populations, procedures for managing algorithm updates or re-training, and post-market monitoring plans that detect performance drift [13,31,67]. For POC diagnostics, this implies (a) demonstrating algorithm generalizability across intended specimen types, collection practices and users; (b) documenting robustness to domain shifts (different geographies, demographic mixes, device cameras/readers); and (c) committing to ongoing surveillance and governance procedures for retraining, data governance, and user notification [13,31,66,67].

Manufacturers should therefore treat AI as a regulated subsystem of the IVD: premarket submissions must include algorithm performance metrics, training/validation data provenance, bias mitigation strategies, and a risk-based plan for post-market monitoring and updates. Health Canada and EU authorities are issuing parallel guidance that will be incorporated into clinical evidence expectations under their respective frameworks; WHO PQDx will likely consider AI readiness under TPP and usability criteria for low-resource settings [59,60,61,62,63,64,65].

### 6.3. Implementation and Adoption

Regulatory clearance is necessary but not sufficient for successful adoption of POC tests. Clearance/waiver enables deployment but does not guarantee clinical uptake, which depends on an integrated implementation plan addressing training, supply chain robustness, connectivity, quality assurance, reimbursement, and stakeholder engagement [67,68,69,70].

Training & competency. End-user training must be tailored to the target operator group (nurses, pharmacists, community health workers, or patients). Critical competencies include correct specimen collection, adherence to IFU steps (timing, buffer volumes, device handling), interpretation of borderline or invalid results, quality control execution, and escalation/reflex algorithms for ambiguous results. Training programs should combine job aids, competency checklists, and periodic proficiency assessments.

Supply chain and quality. Reliable supply chains maintain test quality and consistent performance: lot-to-lot consistency, stable cold-chain or ambient-stability logistics where relevant, timely delivery of controls/calibrators, and planned mitigation for shortages (secondary suppliers, validated alternative lots). Supply failures or degraded reagents directly degrade field performance and can undermine confidence and surveillance systems [66,67,68].

Connectivity & surveillance. Integration with electronic health records (EHRs), laboratory information systems (LISs), and public health reporting is increasingly essential, especially for outbreak-prone pathogens. Connected POC devices enable automatic case notification, geotagged incidence mapping, automated quality-control telemetry, and rapid identification of performance anomalies across networks; these capabilities both improve situational awareness and support regulatory post-market surveillance [65,66].

Quality assurance & governance. Facilities running POC testing should follow CLSI/CAP/CLIA guidance for quality management, including use of controls, participation in external quality assessment (EQA) programs, device maintenance schedules, and incident reporting protocols. Manufacturers and health systems must align on QA responsibilities and reporting workflows prior to large-scale deployment [67].

Reimbursement & economic drivers. POC adoption is strongly influenced by reimbursement policies and demonstrated health-economic value. Publication of cost-effectiveness or implementation studies showing reduced length of stay, antibiotic stewardship benefits, and avoided downstream costs is often required to secure payer coverage and institutional adoption [68].

Stakeholder engagement & implementation science. Successful roll-out benefits from early engagement with laboratory directors, clinicians, payers, and end-user communities. Implementation science tools (workflow mapping, pilot studies, cost-utility analyses) help demonstrate real-world value beyond analytic accuracy [69,70].

In summary, while regulatory clearance and CLIA waiver are important enabling steps, sustainable adoption of POC infectious disease diagnostics requires a comprehensive operational plan (training, supply chains, connectivity, QA, reimbursement and stakeholder engagement) that translates validated performance into improved clinical and public health outcomes [66,67,68,69,70].

## 7. Public Health Considerations

Point-of-care (POC) infectious disease diagnostics play a critical role at the population level, extending beyond individual clinical benefits to shape disease control, surveillance, and health-system efficiency (Table 5). Their most immediate impact lies in reducing diagnostic delays and ensuring that effective treatment and public health actions can begin at the first point of contact.

Community and home testing for HIV have demonstrably increased testing uptake, earlier linkage to antiretroviral therapy (ART), and reduced loss-to-follow-up. Community-based and self-testing programs lower access barriers (geographic, social, and stigma-related), permit repeat testing without facility visits, and enable rapid linkage pathways that improve retention in care, especially in low-resource settings where centralized laboratory access is limited [46,54]. These programs, therefore, contribute directly to earlier diagnosis and treatment initiation and help close testing gaps in underserved populations.

POC molecular platforms for tuberculosis (TB), notably cartridge systems deployed at peripheral clinics, have transformed case detection by enabling same-visit diagnosis and earlier treatment initiation. Earlier diagnosis reduces the infectious period of untreated TB cases, thereby lowering transmission risk in communities and facilitating more rapid public health contact tracing and treatment support [39,62]. Field evidence indicates that decentralized NAAT-based diagnosis shortens time-to-treatment and can improve case notification completeness in high-burden settings.

Integration with digital systems amplifies POC value. Smartphone-coupled readers and cloud-based platforms allow near real-time reporting of test results, geospatial incidence tracking, and automated aggregation of quality-control telemetry, capabilities that strengthen outbreak surveillance and antimicrobial-resistance monitoring when linked to public health databases [50]. WHO and CDC initiatives increasingly emphasize the integration of POC-generated data into national surveillance systems to support earlier warning and faster responses [16,58,65].

Equity and implementation gaps. Despite clear benefits, equitable deployment remains a major challenge. Low- and middle-income countries (LMICs) commonly face barriers in procurement, supply-chain stability, sustainable financing, workforce training, and digital infrastructure, constraints that limit the public health impact of POC innovations and risk widening global inequities in diagnostic access [16,58,65,68]. Targeted investments in supply-chain resilience, connectivity, local capacity building, and financing mechanisms are therefore essential to ensure that POC advances translate into globally distributed public health gains.

In summary, POC diagnostics function not only as clinical tools but as public health enablers: they reduce time-to-diagnosis and treatment, improve outbreak control capacity, support antimicrobial stewardship, and can extend diagnostic access to underserved communities. Realizing their full population-level benefits requires deliberate integration with health-system workflows, robust supply chains, digital connectivity, and sustained financing, particularly in resource-limited settings.

## 8. Future Directions and Gaps

The point-of-care (POC) infectious disease diagnostics field continues to evolve rapidly, driven by convergence across artificial intelligence (AI), miniaturized biosensing, and connected health ecosystems. Yet, several structural gaps (spanning validation, regulatory harmonization, and equitable access) remain to be addressed.

### 8.1. Artificial Intelligence and Digital Integration

Artificial intelligence (AI) and digital connectivity are poised to transform POC testing. Machine learning (ML)–enabled readers and smartphone-based platforms can automate result interpretation, enhance sensitivity, and quantify assays traditionally considered qualitative. For instance, ML algorithms applied to lateral-flow immunoassay images have improved specificity from ~89% to 100% and sensitivity from ~95.6% to ~97.8% compared with human interpretation [71]. Similarly, smartphone-integrated deep-learning systems for SARS-CoV-2 detection have achieved diagnostic accuracies exceeding 98% across diverse users [72]. Emerging AI-driven verification algorithms now allow adaptive real-time interpretation, reducing assay run times from 15 min to as little as 2 min in prototype systems, without sacrificing diagnostic accuracy [73].

Future POCTs are likely to incorporate on-device AI for automated quality control, cloud-assisted multiplex interpretation, and secure real-time data transmission to public health databases. However, these advances introduce new challenges: regulatory frameworks for AI-enabled IVDs remain in development, and manufacturers must address data privacy, algorithmic bias, lifecycle monitoring, and performance drift across diverse populations [74]. Establishing standardized metrics for AI explainability, reproducibility, and fairness will be essential for sustainable adoption and regulatory confidence.

### 8.2. Global Harmonization and Standards

Despite recent progress through the WHO Global Model Regulatory Framework and IMDRF initiatives, a lack of harmonized performance and validation standards for POC diagnostics persists. Many low- and middle-income countries (LMICs) lack specific POCT approval pathways, resulting in dependence on imported devices with limited local validation or post-market oversight [75]. Building regional regulatory capacity, strengthening WHO PQDx laboratories, and developing reference centers capable of independent verification are essential steps toward ensuring diagnostic equity and consistent quality across geographies.

### 8.3. Post-Market Surveillance

Post-market surveillance (PMS) is shifting from periodic reporting toward continuous, data-driven performance monitoring. Regulatory bodies under the FDA and EU IVDR now require real-world performance tracking, risk management systems (ISO 14971), and periodic safety updates [76]. Modern connected POC devices, with built-in telemetry and cloud connectivity, offer opportunities for automated field surveillance, early detection of performance drift, and feedback loops to manufacturers and regulators. However, published post-market evidence remains limited, highlighting the need for large-scale, real-world implementation studies that capture operational reliability, usability, and health-system impact.

### 8.4. Novel Technologies and Platforms

CRISPR-based assays such as SHERLOCK and DETECTR represent a major leap forward in molecular POC diagnostics. These platforms combine the high analytical sensitivity and specificity of nucleic acid amplification with simplified, equipment-free workflows, making them well-suited for decentralized and outbreak-response contexts [77]. CRISPR systems detect pathogen genetic material through collateral cleavage mechanisms, producing rapid colorimetric or fluorescent readouts that can be interpreted visually or via smartphone apps. Their modularity allows rapid assay reprogramming for emerging pathogens, an essential feature for pandemic preparedness. 

Parallel advances in microfluidic “lab-on-chip” devices and paper-based multiplex assays promise simultaneous detection of multiple pathogens from a single specimen [78]. The convergence of CRISPR detection with microfluidics and digital readers could enable next-generation POC systems offering laboratory-level accuracy in compact, low-cost formats.

### 8.5. Economic and Equity Considerations

Economic models and reimbursement frameworks continue to lag behind technical innovation. In many jurisdictions, centralized laboratory testing remains the default reimbursable model, constraining POC uptake [10,20]. Integration of POC diagnostics into value-based care models (where reduced hospitalizations, earlier therapy initiation, and improved patient satisfaction are recognized as cost offsets) is urgently needed.

Moreover, equitable access remains a global challenge [72]. Persistent barriers such as fragmented procurement, inconsistent supply chains, and limited user training hinder deployment in LMICs. Coordinated strategies among manufacturers, policymakers, and funders are required to support local manufacturing, regulatory agility, and inclusive implementation planning [3,10].

In summary, the future of POC infectious disease diagnostics will be defined by intelligent, connected, and modular systems that blend molecular sensitivity with digital precision. Achieving this vision demands harmonized regulations, continuous real-world monitoring, equitable access, and reimbursement reform to ensure that innovation translates into global health impact.

## 9. Discussion

The landscape of point-of-care (POC) infectious disease diagnostics is evolving rapidly, driven by technological innovation, regulatory adaptation, and expanding clinical and public health needs. The preceding sections outlined global market dynamics, technology platforms, validation frameworks, and regulatory guidance. Here we synthesize those insights to clarify how POC diagnostics are positioned to expand their role in infectious disease management and what practical requirements must be met to realize that potential.

### 9.1. Market–Technology Integration

The global POC infectious disease diagnostics market is projected to nearly double between 2024 and 2033, with North America continuing to dominate overall revenues [1,2]. Immunoassays remain the largest segment by volume because of affordability and accessibility, while molecular platforms are expanding rapidly because of superior sensitivity and multiplexing capabilities [3,4]. Biosensors and microfluidic devices, though currently smaller segments, represent key growth areas, particularly given their strong synergy with artificial intelligence (AI) integration [5,13]. AI should be regarded as a transversal enabler rather than a separate market category: it will be embedded into immunoassays (smartphone image readers), molecular assays (algorithmic signal deconvolution), biosensors (machine-learning denoising and pattern recognition), and microfluidic systems (automated interpretation and closed-loop device control). This hybrid framework positions AI as a cross-cutting performance and usability enhancer, consistent with evolving regulatory guidance [6,7].

### 9.2. Validation–Regulation Pipeline

**Figure 3:**
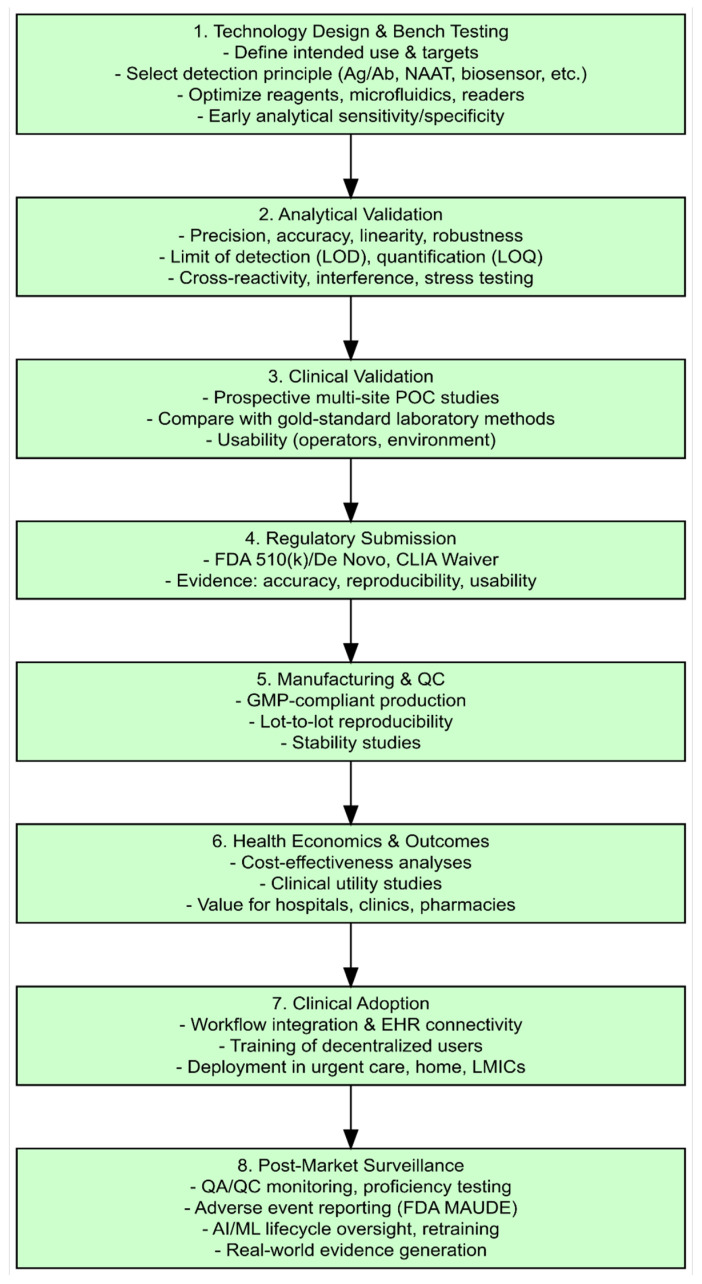
***Validation and implementation pipeline for point-of-care (POC) infectious disease diagnostics.*** The flowchart outlines the sequential stages from assay design and bench validation through analytical and clinical performance studies, regulatory submission (e.g., FDA clearance with potential CLIA waiver), reimbursement and guideline integration, to post-market surveillance. AI intersects multiple stages, including validation (algorithm training), regulatory review (FDA lifecycle guidance), and post-market oversight. Adapted from published validation frameworks and regulatory guidance [11,12,26,52,57].

The validation → regulation → implementation pipeline (Figure 3) highlights sequential but overlapping stages from bench validation through clinical performance (clinical validation) studies, regulatory submission (including CLIA-waiver considerations in the U.S.), reimbursement and guideline integration, and post-market surveillance. Bench validation emphasizes analytical metrics (limit of detection, precision, linearity, interference), whereas later clinical performance studies must demonstrate consistent sensitivity/specificity across the intended use population and settings; human-factors/usability evidence is increasingly required to support decentralized use claims and CLIA waivers [8,10]. For AI-enabled devices, validation must explicitly include algorithm training on diverse datasets, external validation cohorts, and pre-specified monitoring metrics to detect drift after deployment [13,75].

Prevalence and interpretation. Prevalence in the tested population critically modifies the practical performance of any diagnostic test because predictive values depend on pretest probability. Sensitivity and specificity are intrinsic properties of a test; positive predictive value (PPV) and negative predictive value (NPV) are functions of sensitivity, specificity, and prevalence. For example (illustrative only): in a population with 1% prevalence, a test with 80% sensitivity and 98% specificity yields approximately PPV ≈ 29% and NPV ≈ 99.8% (i.e., a negative result is highly reassuring, but a positive result has a high false-positive rate in low-prevalence settings). By contrast, at 20% prevalence, the same test produces a PPV ≈ 91% and an NPV ≈ 95%, so positives are more reliable, but false omissions (false-negative risk) increase. These effects mean that test selection and intended use must be matched to prevalence; screening low-prevalence populations favors inexpensive, high-NPV approaches with reflex confirmatory testing for positives, while diagnostic testing in higher-prevalence clinical settings requires higher sensitivity to minimize false omissions. Diagnostic reports and clinical guidance should therefore present PPV/NPV (or false omission rates) across relevant prevalence ranges to guide implementation and interpretation [54,79].

Regulatory context and EUA experience. During public health emergencies (e.g., COVID-19), Emergency Use Authorization (EUA) pathways accelerate access but sometimes rely on limited pre-market data. While many EUA-cleared POC NAATs and antigen tests performed well, experience also showed variability in real-world performance and the importance of subsequent independent evaluations and post-market surveillance [19,40]. Consequently, manufacturer submissions and regulatory evaluation should place emphasis on robust clinical and usability data, and regulators should require (or incentivize)early post-market validation studies and external performance monitoring to detect underperforming products sooner.

### 9.3. Implementation and Adoption

Regulatory clearance alone does not ensure clinical uptake. Adoption typically depends on the alignment of clinical guidelines, reimbursement policies, and health-system integration. Historical examples illustrate this: widespread public health impact of HIV self-tests occurred only after endorsements from major public health bodies and when linkage-to-care pathways and payer mechanisms were clarified [6]. Similarly, molecular POC platforms for respiratory viruses gained routine clinical use in many hospitals after reimbursement codes were established and real-world studies demonstrated reductions in length of stay and improved antimicrobial stewardship [21]. For POC devices to contribute at scale, developers must plan for implementation, training programs, supply-chain resilience, connectivity for reporting, and economic evidence demonstrating value to payers and health systems [66,67,68].

### 9.4. AI Integration in the Pipeline

AI intersects the entire product lifecycle. At validation, AI models must be trained on diverse, representative datasets and assessed on external cohorts that reflect intended real-world variability; at regulatory review, sponsors should describe training data provenance, performance across subgroups, and update/change protocols; and in the post-market phase, automated monitoring must detect performance drift due to population shifts or new pathogen variants [7,16,76]. Robustness, explainability, and fairness metrics should be part of clinical study plans and regulatory submissions.

### 9.5. Public Health

At the population level, the value of POC diagnostics lies in reducing time-to-diagnosis, expanding decentralized access, and enriching surveillance. Table 5 summarizes demonstrated public health impacts (HIV, TB, malaria, COVID-19, influenza/RSV, HCV, and emerging pathogens). Connectivity (smartphone readers and cloud platforms) amplifies POC public health utility by enabling near real-time aggregation of geo-temporal data for outbreak detection and AMR surveillance. Achieving these benefits at scale requires interoperability standards and data governance frameworks to permit secure, privacy-preserving data sharing with public health authorities [63,68].

### 9.6. Synthesis and Gaps

The POC diagnostics ecosystem is moving toward digitized, AI-enabled, and (potentially) harmonized global systems. Yet the following key gaps constrain impact:

- Heterogeneous validation across populations (LMICs, pediatric, and immunocompromised) limits generalizability.
- Incomplete AI regulatory harmonization across jurisdictions complicates multinational deployment.
- Unclear reimbursement pathways impede clinical adoption despite demonstrable system value.
- Fragmented integration with surveillance systems reduces the population-level utility of POC data streams.

Importantly, the practical success of POC diagnostics depends less on isolated analytical breakthroughs than on coordinated efforts spanning engineering, clinical evaluation, regulatory strategy, reimbursement planning, and implementation science. Only by addressing these interdependent elements will POC technologies translate technical potential into measurable health impacts.

## 10. Conclusions

Point-of-care (POC) infectious disease diagnostics are entering a decisive decade of transformation. The convergence of molecular amplification, biosensors, microfluidics, and artificial intelligence (AI) is reshaping how infectious diseases are detected and managed across clinical and community settings. These innovations extend far beyond technological novelty; they represent a paradigm shift toward decentralized, data-connected, and patient-centered diagnostics.

Moving forward, progress will depend on three interdependent pillars.

**First**, rigorous analytical, clinical, and usability validation must remain the cornerstone of innovation. The COVID-19 pandemic revealed how test performance can vary dramatically with disease prevalence and user context; future evaluations must therefore incorporate real-world, prevalence-adjusted performance metrics and transparent reporting.

**Second**, adaptive regulatory and reimbursement frameworks are essential. The FDA’s lifecycle guidance for AI-enabled devices and the European IVDR’s emphasis on continuous performance monitoring illustrate a global shift toward dynamic, evidence-based oversight. Alignment across agencies and health systems will be key to accelerating safe and equitable adoption.

**Third**, the public health dimension must not be secondary. True impact arises when validated POC tests strengthen surveillance, antimicrobial stewardship, and outbreak response, especially in resource-limited settings where diagnostic access remains a major barrier.

The coming decade will likely see POC diagnostics evolve from rapid testing tools into integrated components of intelligent, connected health ecosystems. If scientific rigor, regulatory agility, and implementation equity advance together, POC technologies can meaningfully enhance clinical care, strengthen health systems, and reinforce global preparedness against future infectious threats.

## Figures and Tables

**Table 1:** Estimated AI integration in point-of-care (POC) infectious disease diagnostic technologies, 2024–2034 [30,31,32,33].

Technology	Estimated % AI-Enabled in 2024	Projected % AI-Enabled in 2034	Rationale
Immunoassays (LFAs)	3%	15%	AI mainly via smartphone readers/image analysis and QC; adoption is slower for simple strips but rising with digital readers.
Molecular (POC NAAT/PCR)	7%	35%	Molecular platforms already have software; AI can assist interpretation, QC, and multiplex deconvolution: faster uptake expected.
Biosensors	8%	40%	Biosensors often produce signals that benefit from ML denoising and pattern detection: strong AI synergy.
Microfluidics	10%	45%	Microfluidic lab-on-chip platforms with integrated sensors and data streams are prime targets for embedded AI.
Others	2%	10%	Miscellaneous devices may incorporate niche AI features (e.g., algorithm-assisted interpretation in niche platforms).

**Table 2:** **Comparative characteristics of point-of-care (POC) diagnostic platforms for human infectious diseases.** Summary of detection principles, sample types, typical biomarkers, representative commercial devices, time-to-result, and major strengths and limitations across immunoassays, molecular platforms, biosensors, microfluidics, and emerging technologies [34,35,36,37,38,39,40,41,42,43,44,45,46,47,48].

Technology	Detection Principle	Typical Sample Types	Common Targets/Biomarkers	Representative Commercial Examples *	Time-to-Result	Strengths	Limitations
**Immunoassay (Lateral flow; reader-assisted FIA)**	Antigen–antibody binding; colorimetric or fluorescent readout	Nasal/nasopharyngeal swab, oral fluid, fingerstick blood/serum/plasma	Viral antigens (SARS-CoV-2, Influenza, RSV), bacterial antigens (Strep A), host antibodies (HIV, HCV), parasite antigens (malaria)	Abbott BinaxNOW (COVID-19, Influenza), BD Veritor (Flu/RSV/COVID), Quidel Sofia 2 (Flu/RSV/COVID/Strep), OraQuick HIV-1/2, OraQuick HCV	~10–30 min	Low cost; simple workflow; CLIA-waived/OTC options; huge installed base	Lower analytical sensitivity vs. NAAT; performance varies with viral load and collection; limited multiplexing (except some reader-based FIAs)
**Molecular (NAAT: PCR/RT-PCR; isothermal)**	Nucleic-acid amplification and detection (optical/fluorescent)	Swabs, urine, whole blood/plasma, vaginal/cervical swabs	Pathogen genomes (SARS-CoV-2, Flu/RSV, CT/NG, TB, HIV RNA)	Cepheid Xpert Xpress (Flu/RSV/COVID), Roche cobas Liat (Flu/RSV/COVID/Strep), Abbott ID NOW (Flu/COVID/Strep), Visby Medical Sexual Health (CT/NG/TV), binx io CT/NG	~13–60 min	High clinical sensitivity/specificity; closed-cartridge “sample-in/answer-out”; multiplex panels	Higher cost; some systems moderate complexity; supply/logistics for single-use cartridges
**Biosensors (electrochemical/optical)**	Biorecognition (antibody/aptamer/NA) transduced to electrical/optical signal	Fingerstick whole blood/serum/plasma, saliva, swabs	Antigens, antibodies, short nucleic-acid targets	Reader-based immunosensors (e.g., handheld fluorimeters/electrochemical meters powering branded rapid tests), smartphone-coupled readers	~5–30 min	Quantitation possible; small readers; connectivity/AI-friendly	Fewer FDA-cleared infectious examples than LFA/NAAT; calibration & drift; often single-analyte
**Microfluidics (lab-on-chip/cartridge)**	Integrated sample prep, metering, reactions, detection in sealed cartridge	Whole blood, swabs, urine, saliva	Multiplex antigen/NA panels; host biomarkers	Many NAAT cartridges and some immunoassay cassettes are microfluidic under the hood (e.g., Liat, Visby, Xpert)	~10–60 min	Automates multi-step workflows; reduces contamination; supports multiplexing	Device/cartridge cost; waste; thermal/valving complexity
**Others (emerging/adjacent)**	CRISPR readouts; portable sequencing; phone-based optics	Swabs, blood, saliva	Genetic targets; broad metagenomics	CRISPR strip/reader prototypes; Oxford Nanopore MinION (surveillance/near-POC)	~20–60+ min (var.)	Ultra-sensitive or broad target scope; future-proof	Limited FDA-cleared POC use to date; workflow standardization maturing

* Examples are illustrative, not exhaustive; availability, indications, and complexity status vary by country and time.

**Table 3:** **Representative FDA-cleared or CLIA-waived POC infectious disease diagnostics in the United States.** Selected examples of antigen, molecular, and antibody-based assays, showing disease target, representative products, typical sample, time-to-result, and accuracy metrics as reported in FDA summaries and peer-reviewed evaluations [52,53,54,55,56,57,58,59].

Disease/Syndrome	POC Method	Representative U.S. Products (FDA Cleared; Many CLIA-Waived) **	Typical Sample	Time-to-Result	Accuracy (Qualitative, per IFU/Comparator)
**COVID-19**	Antigen (LFA/FIA)	Abbott BinaxNOW COVID-19 Ag Card; Quidel Sofia 2 SARS Antigen FIA; BD Veritor SARS-CoV-2	Nasal swab	10–20 min	Moderate–High (load-dependent)
	NAAT (PCR/Isothermal)	Cepheid Xpert Xpress SARS-CoV-2; Roche cobas Liat SARS-CoV-2; Abbott ID NOW COVID-19	Nasal/NPS swab	13–45 min	High
**Influenza A/B (± RSV)**	Antigen (FIA)	Quidel Sofia 2 Flu + SARS Antigen; BD Veritor Flu A+B; Abbott BinaxNOW Influenza	Nasal/NPS swab	10–20 min	Moderate–High
	NAAT (PCR)	Roche cobas Liat Influenza A/B (± RSV); Cepheid Xpert Xpress Flu/RSV	Nasal/NPS swab	20–45 min	High
**Group A Streptococcus**	Antigen (LFA/FIA)	Quidel QuickVue Strep A; BD Veritor Strep A; Abbott Strep A tests	Throat swab	5–15 min	Moderate–High (culture confirm often recommended if negative)
	NAAT (isothermal/PCR)	Abbott ID NOW Strep A; Roche cobas Liat Strep A	Throat swab	6–20 min	High
**HIV-1/2 (antibody/Ag-Ab)**	Rapid immunoassay	OraQuick ADVANCE Rapid HIV-1/2 Ab; Determine HIV-1/2 Ag/Ab Combo ***	Fingerstick whole blood, oral fluid (OraQuick)	20–30 min	High (oral-fluid sensitivity slightly lower vs. serum)
**Hepatitis C (HCV)**	Rapid antibody test	OraQuick HCV Rapid Antibody Test	Fingerstick whole blood/serum/plasma	~20 min	High (antibody only; RNA confirm required)
**CT/NG (Chlamydia/ Gonorrhea)**	NAAT (POC)	binx io CT/NG; Visby Medical Sexual Health (CT/NG/TV)	Vaginal/urethral swab; urine (per IFU)	~30 min (binx)/~30–40 min (Visby)	High
**RSV**	Antigen (FIA)	Quidel Sofia 2 RSV; BD Veritor RSV	Nasal/NPS swab	10–15 min	Moderate–High
**Malaria**	Rapid antigen test	BinaxNOW Malaria	Whole blood (fingerstick)	~15 min	High for *P. falciparum* HRP-2; lower for non-falciparum antigens

Table Notes: **Regulatory/complexity status can change. Many listed assays are CLIA-waived for specific specimen types; always confirm current FDA/CLIA status and IFU details before citing. *** Some combo Ag/Ab HIV rapid tests are cleared for professional use; CLIA-waiver and oral-fluid use vary by product/setting. “Accuracy” is summarized qualitatively (e.g., “High” for NAAT). Specific PPA/NPA depend on study design, specimen, lineage, and comparator.

**Table 4:** Comparison of regulatory pathways for POC infectious disease diagnostics: FDA (U.S.), Health Canada, EU (IVDR), and WHO Prequalification (PQDx). Summary items correspond to the text and authoritative guidance [7,57,58,59,60,61,62,63,64,65,66,67].

Feature	FDA (U.S.)	Health Canada	EU (IVDR)	WHO Prequalification (PQDx)
**Regulatory authority/legal basis**	FDA; Center for Devices & Radiological Health (CDRH). IVDs cleared via 510(k), De Novo, or PMA; CLIA categorization determines “waived” status.	Health Canada; Medical Device Directorate; devices licensed via Medical Device Licence (MDL) for Class II–IV. Guidance documents for near-patient devices.	EU Regulation 2017/746 (IVDR); conformity assessment by Notified Bodies; stricter clinical evidence and post-market requirements vs. IVDD.	WHO PQDx: voluntary prequalification for priority IVDs (HIV, malaria, viral load, etc.) focused on suitability for low-resource settings; used for UN procurement.
**Risk classification (typical for POC IVDs)**	IVDs range Class I–III by risk; many POC infectious tests treated as moderate-risk (Class II). CLIA complexity categories (waived/moderate/high) differ from FDA device class.	Risk-based rules (Schedule I) classify IVDs I–IV; near-patient often Class II or higher depending on intended use.	IVDR risk classes A–D (D highest). Many clinically actionable POC infectious IVDs fall into Class B or C under IVDR (increased clinical evidence required).	PQ focuses on priority disease targets (not a national regulatory approval). Devices must meet WHO technical specifications/TPPs for intended use.
**Analytical evidence required**	LoD, precision, linearity, interfering substances, cross-reactivity, stability studies; documented per FDA guidance.	Similar analytical dossier; Health Canada requests validation data proportional to classification.	IVDR requires performance evaluation report (analytical performance) and supporting documentation to Notified Body.	PQ requires analytical validation demonstrating fitness for intended use in target contexts (stability, matrix effects).
**Clinical evidence required**	Clinical performance compared to reference methods (sensitivity/specificity, PPA/NPA) in intended-use populations; CLIA-waiver submissions often require usability/clinical studies.	Clinical evidence proportional to risk class; Health Canada expects data or justification with equivalent evidence.	IVDR requires clinical performance studies and evidence in intended populations.	PQ expects clinical/field evaluation evidence in intended settings, often prospective field studies.
**Human factors/usability studies**	Required for CLIA-waiver and home-use claims; FDA provides guidance for usability and labeling.	Requested where relevant to demonstrate safe near-patient use.	IVDR/MDCG emphasize usability and labeling; may form part of clinical evidence.	PQ evaluates ease-of-use and suitability for LMICs (training needs, storage, stability).
**POC-specific criteria (e.g., CLIA waiver)**	CLIA-waiver requires demonstration of simplicity and low error risk; OTC/home-use tests automatically waived.	No CLIA-equivalent; Health Canada may authorize near-patient use with labeling and risk mitigation.	CE-marking under IVDR permits POC use as per IFU; no waiver scheme equivalent to CLIA.	PQDx evaluates POC suitability via TPPs and field performance; used for procurement.
**Post-market surveillance & vigilance**	PMS under 21 CFR 820, MDR reporting, MAUDE adverse event reporting. FDA also piloting AI/ML lifecycle oversight.	Vigilance reporting and MDL maintenance; proportional to device risk.	IVDR mandates PMS, UDI, periodic safety update reports.	PQ requires ongoing monitoring and periodic reassessment for continued eligibility.
**Typical timelines/practical notes**	510(k): months; CLIA-waiver adds human-factors/robustness review. De Novo/PMA longer. EUA faster. Early FDA interaction recommended.	Timelines vary by class and dossier completeness; pre-submission meetings are recommended.	Timelines depend on class and Notified Body capacity; IVDR increases evidence requirements since 2022.	

**Table 5:** Demonstrated public health impact of POC diagnostics for major infectious diseases [7,30,39,50,53,55,61,62,63,64,65,66].

Infectious Disease	POC Diagnostic Type	Demonstrated Public Health Impact
**HIV**	Rapid antibody/Ag-Ab tests (e.g., OraQuick, Determine Combo)	Increased testing uptake in community and home settings; earlier ART initiation; reduced loss-to-follow-up in LMICs
**Tuberculosis (TB)**	Molecular NAAT (e.g., GeneXpert, Truenat)	Faster diagnosis and treatment initiation; reduced transmission; improved case detection in peripheral clinics
**Malaria**	Rapid antigen tests (HRP2-based RDTs)	Expanded access in remote areas; improved case management; major role in WHO test-and-treat strategies
**COVID-19**	Antigen LFAs and rapid NAAT	Enabled mass decentralized screening; informed isolation/quarantine decisions; supported outbreak containment
**Influenza/RSV**	Rapid antigen and molecular assays (e.g., Sofia, Liat)	Improved antimicrobial stewardship; reduced unnecessary antibiotic prescriptions; faster triage in emergency settings
**HCV**	Rapid antibody test (e.g., OraQuick HCV)	Increased community screening; improved linkage-to-care in marginalized populations
**Emerging pathogens** (e.g., Ebola, Zika)	Prototype LFAs, molecular assays	Field-deployed tools during outbreaks; enabled surveillance and case finding in epidemic hotspots

## Data Availability

No new data were created or analyzed in this study. Data sharing is not applicable.

## Abbreviations

AI: Artificial intelligence. AMR: Antimicrobial resistance. ART: Antiretroviral therapy. CAP: College of American Pathologists. CDC: Centers for Disease Control and Prevention. CDRH: Center for Devices and Radiological Health (FDA). CLIA: Clinical Laboratory Improvement Amendments. CLIN: Clinical. CLSI: Clinical and Laboratory Standards Institute. CFR: Code of Federal Regulations (U.S.). CRISPR: Clustered Regularly Interspaced Short Palindromic Repeats. CT/NG/TV: Chlamydia trachomatis/Neisseria gonorrhoeae/Trichomonas vaginalis. EHR: Electronic health record (interpreted correction for “HER”). EUA: Emergency Use Authorization (FDA). EU: European Union. FIA: Fluorescence immunoassay. GMP: Good Manufacturing Practice. HCV: Hepatitis C virus. HIV: Human immunodeficiency virus. HPV: Human papillomavirus. IFU: Instructions for Use. IMDRF: International Medical Device Regulators Forum. IVD: In vitro diagnostic device. IVDR: In Vitro Diagnostic Regulation. (EU) IoMT: Internet of Medical Things. LFA: Lateral flow assay. LOD: Limit of detection. LOQ: Limit of quantitation. LAMP: Loop-mediated isothermal amplification. LMIC: Low- and middle-income country/countries. MDCG: Medical Device Coordination Group (EU). MDL: Medical Device Licence (Health Canada). ML: Machine learning. MAUDE: Manufacturer and User Facility Device Experience (FDA adverse events database). NAAT: Nucleic acid amplification test. NPV: Negative predictive value. OTC: Over-the-counter. PACS: Picture archiving and communication system *)*. PMA: Premarket Approval (FDA). PMS: Post-market surveillance. PPA/NPA: Positive percent agreement/Negative percent agreement. POC: Point-of-care. POCT: Point-of-care testing. PQDx: WHO Prequalification of Diagnostics (PQDx). PCR: Polymerase chain reaction. QC: Quality control. QMS: Quality management system (interpreted correction for “QMAG”). REASSURED: Relevance, Easy, Affordable, Sensitive, Specific, User-friendly, Rapid/Robust, Equipment-free, Delivered (mnemonic for POC). RDT: Rapid diagnostic test. ROC: Receiver operating characteristic. RT-PCR: Reverse-transcription PCR. RSV: Respiratory syncytial virus. SaMD: Software as a Medical Device. SARS-CoV-2: Severe acute respiratory syndrome coronavirus 2. TB: Tuberculosis. TPP: Target product profile. UDI: Unique Device Identifier. WHO: World Health Organization. USD: United States Dollar.
